# A novel lncRNA TCLlnc1 promotes peripheral T cell lymphoma progression through acting as a modular scaffold of HNRNPD and YBX1 complexes

**DOI:** 10.1038/s41419-021-03594-y

**Published:** 2021-03-25

**Authors:** Ping Zhao, Meng-Meng Ji, Ying Fang, Xiao Li, Hong-Mei Yi, Zi-Xun Yan, Shu Cheng, Peng-Peng Xu, Anne Janin, Chao-Fu Wang, Li Wang, Wei-Li Zhao

**Affiliations:** 1 Shanghai Institute of Hematology, State Key Laboratory of Medical Genomics, National Research Center for Translational Medicine at Shanghai, Ruijin Hospital Affiliated to Shanghai Jiao Tong University School of Medicine, Shanghai, China; 2 Department of Pathology, Ruijin Hospital Affiliated to Shanghai Jiao Tong University School of Medicine, Shanghai, China; 3 Pôle de Recherches Sino-Français en Science du Vivant et Génomique, Ruijin Hospital Affiliated to Shanghai Jiao Tong University School of Medicine, Shanghai, China; 4 U1165 Inserm/Université Paris 7, Hôpital Saint Louis, Paris, France

**Keywords:** Non-hodgkin lymphoma, Non-coding RNAs

## Abstract

Long noncoding RNAs (lncRNAs) play an essential role in tumor progression. Few researches focused on the clinical and biological relevance of lncRNAs in peripheral T cell lymphoma (PTCL). In this research, a novel lncRNA (ENST00000503502) was identified overexpressed in the main subtypes of PTCL, and designated as T cell lymphoma-associated lncRNA1 (TCLlnc1). Serum TCLlnc1 was associated with extranodal involvement, high-risk International Prognostic Index, and poor prognosis of the patients. Both in vitro and in vivo, overexpression of TCLlnc1 promoted T-lymphoma cell proliferation and migration, both of which were counteracted by the knockdown of TCLlnc1 using small interfering RNAs. As the mechanism of action, TCLlnc1 directly interacted with transcription activator heterogeneous nuclear ribonucleoprotein D (HNRNPD) and Y-box binding protein-1 (YBX1) by acting as a modular scaffold. TCLlnc1/HNRNPD/YBX1 complex upregulated transcription of *TGFB2* and *TGFBR1* genes, activated the tumor growth factor-β signaling pathway, resulting in lymphoma progression, and might be a potential target in PTCL.

## Introduction

Peripheral T cell lymphomas (PTCL) encompass a heterogeneous group of neoplasm derived from T cell lineages and represent ~10–15% of non-Hodgkin lymphoma^1^, mainly including anaplastic large-cell lymphoma (ALCL)-anaplastic lymphoma kinase (ALK)-positive (ALK^+^ALCL), ALCL-ALK-negative (ALK^−^ALCL), angioimmunoblastic T cell lymphoma (AITL), and PRCL-not otherwise specified (PTCL-NOS)^2^. PTCLs are characterized by advanced disease stage at diagnosis^3^, and dismal prognosis, with a 5-year patients survival rate of <30% (except for ALK^+^ALCL)^4–6^. Therefore, prognostic biomarkers closely related to lymphoma progression and expressed generally in the main histological subtypes need to be further investigated in PTCL.

To date, studies have focused on the alterations in protein-coding genes that serve as diagnostic and therapeutic targets of PTCL^7^. Growing evidence suggests that noncoding RNAs are also critically involved in tumor progression^8,9^. Long noncoding RNAs (lncRNAs) belong to a class of noncoding sequences >200 nucleotides in length^10^ and are emerging as essential factors in the tumorigenesis and metastasis of various cancers, including hematological malignancies^11,12^. The expression of HOTAIR enhances B-lymphoma cell growth through mitogen-activated protein kinase (MAPK) and extracellular signal‐regulated kinase signaling pathway^13^. LncRNA metastasis-associated lung adenocarcinoma transcript 1 is also overexpressed in hematological malignancies^14^, rendering tumor cell resistant to chemotherapeutic agents^15^. As a mechanism of action, lncRNAs exhibit diverse aspects regarding tumor progression through regulating chromatin organization and gene transcription in the nucleus. LncRNAs also modulate messenger RNA (mRNA) stability, translation, and post-translational modification in the cytoplasm^16,17^. However, pathogenic role and underlying molecular mechanism of lncRNAs remain largely unknown in PTCL.

In the present study, we performed genome-wide lncRNA expression assay in PTCL and identified a novel lncRNA (ENST00000503502) associated with lymphoma progression and poor prognosis in patients, designating it as T cell lymphoma-associated lncRNA1 (TCLlnc1). The biological function of TCLlnc1 on T-lymphoma cell proliferation and migration was evaluated in vitro and in vivo.

## Materials and methods

### Patients and clinical specimens

Patients with newly diagnosed, histologically confirmed PTCL treated with cyclophosphamide, doxorubicin, vincristine, and prednisone (CHOP)-based chemotherapy between June 2013 and October 2019 were eligible and enrolled in this study. Exclusion criteria of patients were as previously described^18^. One hundred and thirty-eight PTCL patients were included and their characteristics were summarized in Supplementary Table 1. The study was approved by the Shanghai Ruijin Hospital Review Board with informed consent obtained from all patients in accordance with the Declaration of Helsinki. Pathological diagnosis was established according to the World Health Organization classification^19^.

### Transcriptomic sequencing

Total RNA was extracted from high-quality frozen tumor samples of 40 PTCL cases (from 70 PTCL cases detected for TCLlnc1 levels) using RNA RNeasy Mini Kit (Qiagen, Dusseldorf, German) for RNA sequencing (RNA-seq) and analyzed as previously described^20–25^.

### Cell lines and culture conditions

T-leukemia/lymphoma cell lines Jurkat, Hut78, and embryonic kidney cell line HEK-293T were obtained from the American Type Culture Collection (Manassas, VA, USA) and cultured as previously described^26^.

### Cell cycle assay

Jurkat and Hut78 cells were transfected with TCLlnc1 plasmid or small interfering RNA (siRNA). A total of 1 × 10^6^ Jurkat and Hut78 cells were harvested after 72 h of incubation, stained with propidium iodide (MultiSciences Biotech, Hangzhou, China), and analyzed by flow cytometry^26^.

### Cell proliferation assay

A total of 5 × 10^3^ Jurkat and Hut78 cells were seeded onto 96-well plates per well. After incubation for the indicated time (0, 24, 48, and 72 h), proliferation was measured using Cell Counting Kit-8 (Dojindo, Japan) according to the manufacturer’s protocol^27^.

### Transwell assay

Transwell assay was performed as previously described^28^, the migrated cells in the lower compartments were counted by flow cytometry, and images of the cells fixed on the bottom surface of the filter were taken under the microscope.

### Quantitative real-time PCR

RNA expression levels were measured using quantitative real-time polymerase chain reaction (qRT-PCR) as previously described^26^. Glyceraldehyde-3-phosphate dehydrogenase (*GAPDH*) was used as an endogenous control and Hut78 cells for calibration^29–31^. The primers (Supplementary Table 2) were provided by Biosune Biotechnology Company (Shanghai, China).

### Western blot

Western blot analysis was performed as previously described^32^. Rabbit anti‐heterogeneous nuclear ribonucleoprotein D (HNRNPD) antibody (12382S, Cell Signaling Technology, Danvers, MA, USA) and rabbit anti‐Y-box binding protein-1 (YBX1) (9744S, Cell Signaling Technology) antibody were used for western blot. The dilution of primary antibodies was 1:1000. An anti-GAPDH antibody was used as the endogenous loading control.

### Fluorescence in situ hybridization (FISH)

The expression of TCLlnc1 in Jurkat cells was examined by using in situ hybridization assay^33^, with anti-TCLlnc1 oligodeoxynucleotide probe sets (lncRNA FISH Probe Mix, Sangon Biotech, Shanghai, China).

### In vitro transcription

Full length of TCLlnc1 and antisense TCLlnc1 were cloned from the plasmid of TCLlnc1 into downstream of the T7 promoter using Phanta EVO HS Super-Fidelity DNA Polymerase (Vazyme, Nanjing, China). TCLlnc1 and antisense TCLlnc1 with T7 promoter was purified with Wizard® SV Gel and PCR Clean-Up System (Promega, Madison, WI, USA). In vitro transcription assays were performed using MEGAscript™ T7 Transcription Kit according to the manufacturer’s instructions (Invitrogen).

### RNA pull-down assay

RNA pull-down assays were performed with Pierce™ Magnetic RNA-Protein Pull-Down Kit (Thermo) according to the manufacturer’s instructions^34^.

### RNA immunoprecipitation

RNA immunoprecipitation (RIP) experiments were performed using the Magna RIPTM Kit (Millipore, Billerica, MA, USA). The co-precipitated RNAs were detected by RT-PCR. To demonstrate that the detected RNA signals specifically bind to either HNRNPD or YBX1, the total RNA (input controls) and normal rabbit IgG controls were simultaneously assayed.

### Plasmids, lentivirus construction, and small interfering RNAs

Full-length TCLlnc1 was cloned into the vector pLenti CMV-MCS-EF1α-ZsGreen1-PGK-Puro for overexpression (Lingke Biotech, Shanghai, China). All short hairpin RNA (shRNA) lentivirus and siRNAs involved in the study were constructed by Lingke Biotech. All siRNA sequences are provided in Supplementary Table 3.

### Co-immunoprecipitation (Co-IP)

Manual immunoprecipitation was performed according to Pierce™ Classic Magnetic IP/Co-IP Kit User Guide (Thermo). Both the input and co-IP samples were analyzed by western blot analysis using anti-HNRNPD or anti-YBX1 antibodies (Cell Signaling Technology).

### Luciferase reporter assay

The designated combinations of indicated plasmids and other relevant siRNAs plus pRL-TK-Renilla were transfected into HEK-293T cells with the Lipofectamine 3000 reagent (Invitrogen), and luciferase activity was detected using the Dual-Luciferase Reporter Assay System (Promega).

### In vivo experiments

Female BALB/c nude mice 5 to 6 weeks of age were purchased from the Shanghai Laboratory Animal Center (Shanghai, China). All experiments were performed in accordance with relevant institutional and national guidelines and the regulations of the Shanghai Medical Experimental Animal Care Commission. The subject was approved by the Laboratory Animal Ethics Committee of Ruijin Hospital affiliated with Shanghai Jiao Tong University School of Medicine. In vivo experiments were performed as previously described^26^.

### Statistical analysis

All statistical analyses were performed using SPSS 21.0 (IBM, Armonk, NY, USA). The significance of the differences between groups was estimated using either the Student’s *t* test, *χ*^2^ test, or Mann–Whitney *U* test. Progression-free survival (PFS) and overall survival (OS) were calculated using the Kaplan–Meier method with the log-rank test for comparisons. The Cox regression and proportional hazards models were used for univariate/multivariate analysis. A value of *P* < 0.05 indicated a significant difference.

## Results

### LncRNA candidate ENST00000503502 is clinically relevant in PTCL

**Fig. 1:**
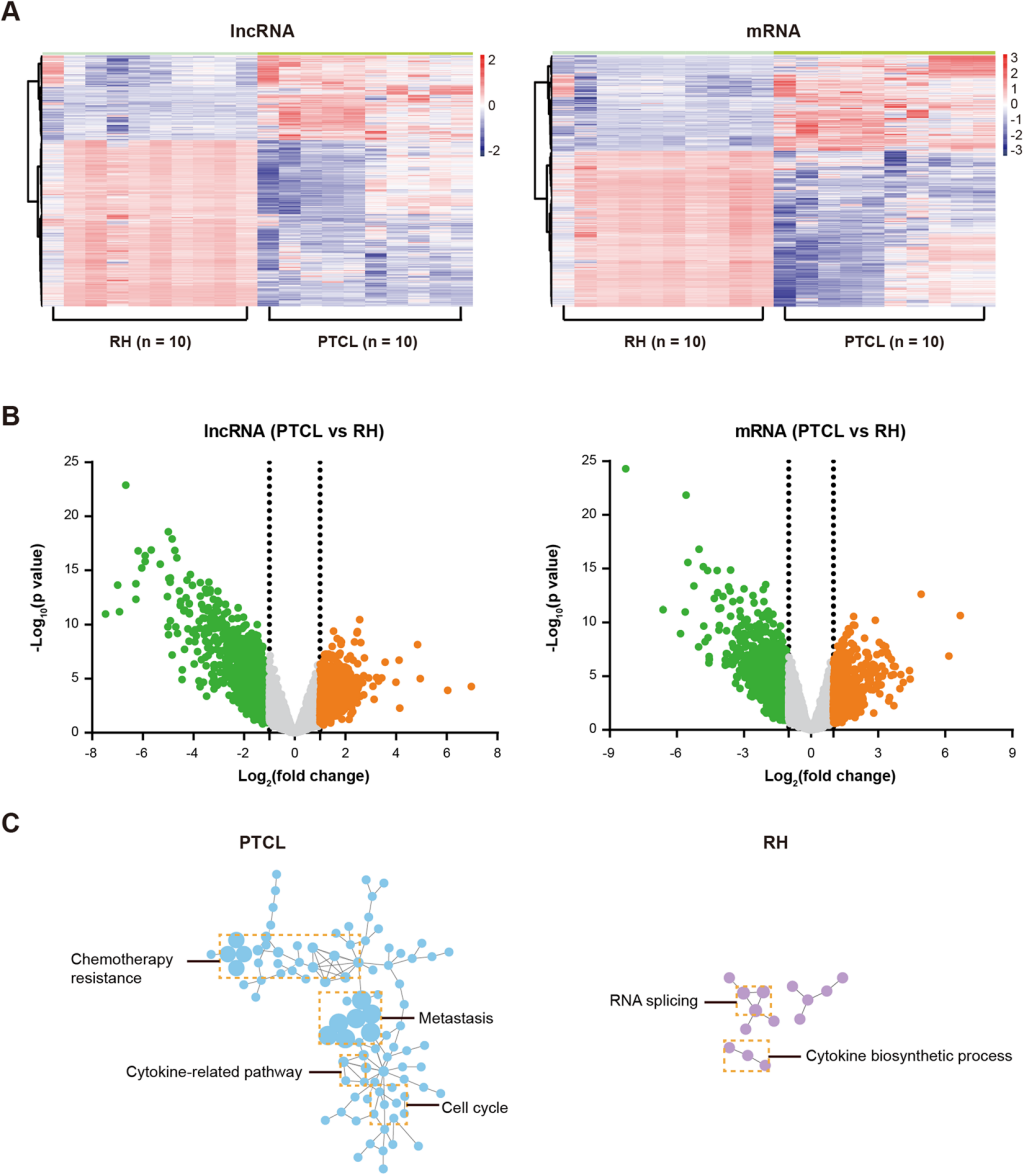
ENST00000503502 was overexpressed and related to oncogenes in peripheral T cell lymphoma (PTCL). **A** LncRNA and mRNA expression in reactive hyperplasia (RH, *n* = 10) and peripheral T cell lymphoma (PTCL, *n* = 10) as revealed by lncRNA and mRNA microarray. **B** Volcano plot images of differentially expressed genes in PTCL and RH. **C** The gene signatures of ENST00000503502-coexpressed mRNAs in PTCL and RH.

LncRNA expression profile was examined in tissue samples of ten PTCL and ten reactive hyperplasia (RH) using Arraystar Human LncRNA Microarray v2.0. In total, 33,045 lncRNAs and 30,215 mRNAs were collected from the authoritative databases RefSeq, UCSC Known Genes dataset, Ensembl, and related literatures, as per the manufacturer’s instructions. Heatmap displayed differentially expressed lncRNAs and mRNAs in PTCL, as compared to RH (Fig. 1A). To identify potential oncogenic lncRNAs, more stringent filtering criteria (raw signal intensity >2000, fold change >2, *P* < 0.01, 200 nt < RNA length < 1000 nt, Fig. 1B) were used and 54 upregulated lncRNAs were obtained; lncRNA-mRNA co-expression network analysis was performed using the Cytoscape software. The top five lncRNAs (ENST00000394174, ENST00000432567, ENST00000503502, ENST00000456305, and NR_033390) with more co-expressed oncogenes (according to the Catalogue Of Somatic Mutations In Cancer [COSMIC, https://cancer.sanger.ac.uk] database) in PTCL than in RH were identified as candidate lncRNAs and assessed by qRT-PCR (Supplementary Fig. 1A). As shown in Supplementary Fig. 1B, ENST00000503502 expression level was significantly higher in PTCL tissues compared with RH tissues (*P* = 0.0004). Thus, among the candidate lncRNAs, ENST00000503502 presented a significant increase in PTCL and had the strongest correlation with the expression of oncogenes, which mainly involved in the cell cycle, cytokine-related pathways, metastasis, and chemotherapy resistance (Fig. 1C); thereby, ENST00000503502 was designated TCLlnc1, and further exploration of clinical significance and biological functions should be explored. TCLlnc1 was located in chromosome 4 (chr4:139,546,266–139,546,727) with a 462 nt length and identified as a lncRNA rather than a protein-coding transcript by CPC (Coding Potential Calculator, http://cpc.cbi.pku.edu.cn). An analysis was performed on the coding potential using CPAT (Coding Potential Assessment Tool, http://lilab.research.bcm.edu/cpat), and the results indicated that TCLlnc1 was unlikely to encode any protein product (Supplementary Fig. 1C).

**Fig. 2:**
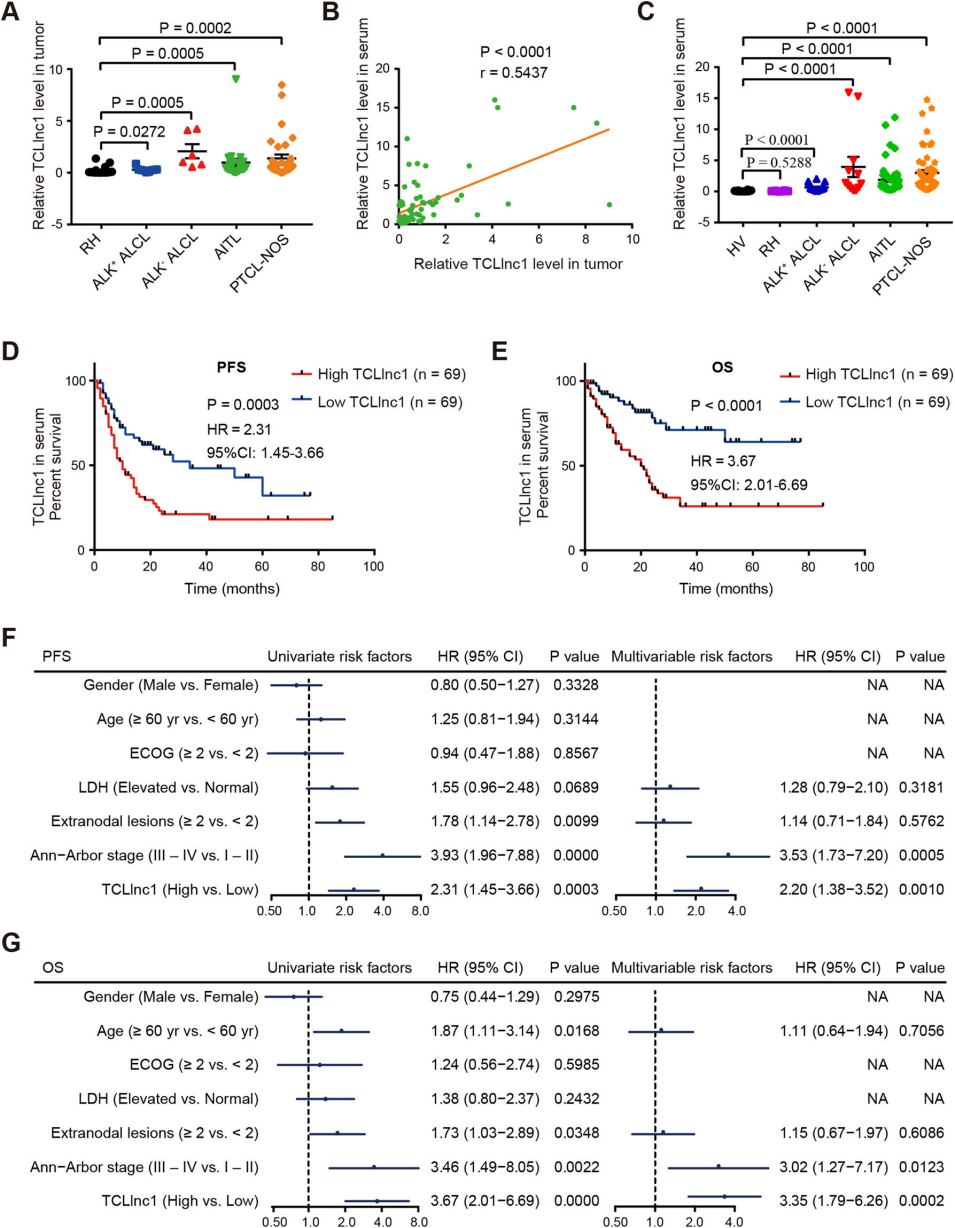
TCLlnc1 was clinically relevant in PTCL. **A** Expression of TCLlnc1 in tumors of anaplastic large-cell lymphoma (ALCL)-anaplastic lymphoma kinase (ALK)-positive (ALK^+^ALCL, *n* = 9), ALCL-ALK negative (ALK^−^ALCL, *n* = 6), angioimmunoblastic T cell lymphoma (AITL, *n* = 23), and peripheral T cell lymphoma-not otherwise specified (PTCL-NOS, *n* = 32), as compared to RH (*n* = 16) by quantitative real-time polymerase chain reaction (qRT-PCR). **B** Correlation between TCLlnc1 expression in tumor and in serum of PTCL patients (*n* = 70). **C** Expression of TCLlnc1 in serum of RH (*n* = 10), ALK^+^ALCL (*n* = 18), ALK^−^ALCL (*n* = 12), AITL (*n* = 53), and PTCL-NOS (*n* = 55) in comparison with healthy volunteers (HV, *n* = 10). TCLlnc1 expression was quantified by qRT-PCR. **D**, **E** Progression-free survival (PFS, **D**) and overall survival (OS, **E**) of PTCL patients according to TCLlnc1 expression in serum (*n* = 138). **F**, **G** Univariate and multivariate analysis for PFS (**F**) and OS (**G**) in PTCL (*n* = 138).

In order to validate the microarray data, TCLlnc1 expression was detected by qRT-PCR in tumor samples of 70 PTCL patients, including 9 cases of ALK^+^ALCL, 6 cases of ALK^−^ALCL, 23 cases of AITL, and 32 cases of PTCL-NOS, as well as 16 cases of RH. Compared with RH (0.224 ± 0.101), the expression level of TCLlnc1 was significantly increased in the main subtypes of PTCL (ALK^+^ALCL, 0.292 ± 0.077, *P* = 0.0272, ALK^−^ALCL, 2.075 ± 0.682, *P* = 0.0005, AITL, 0.982 ± 0.379, *P* = 0.0005, and PTCL-NOS, 1.386 ± 0.365, *P* = 0.0002, Fig. 2A). Moreover, the expression of TCLlnc1 in serum positively correlated with TCLlnc1 in tumors of PTCL patients (*P* < 0.0001, Fig. 2B). TCLlnc1 expression was then assessed in enlarged serum samples of 138 PTCL patients, including 18 cases of ALK^+^ALCL, 12 cases of ALK^−^ALCL, 53 cases of AITL, and 55 cases of PTCL-NOS, as well as in 10 cases of RH and 10 cases of healthy volunteers (HVs). Serum TCLlnc1 was significantly higher in PTCL patients (ALK^+^ALCL, 0.651 ± 0.134, *P* < 0.0001, ALK^−^ALCL, 3.940 ± 1.625, *P* < 0.0001, AITL, 1.879 ± 0.335, *P* < 0.0001, and PTCL-NOS, 2.986 ± 0.457, *P* < 0.0001) than in HV (0.076 ± 0.030), no significant difference was observed between RH and HV (*P* = 0.5288, Fig. 2C).

As shown in Table 1, 138 PTCL patients were divided into high- and low-expression groups based on the median value of TCLlnc1. High TCLlnc1 expression group was significantly associated with extranodal involvement (*P* = 0.0152), high-risk International Prognostic Index (IPI) (*P* = 0.0063), and poor chemotherapy response (*P* = 0.0006). Accordingly, median PFS and OS of the patients in the high TCLlnc1 expression group were 10 and 20 months, respectively; significantly shorter than those of the low TCLlnc1 expression group (34 months, *P* = 0.0003, Fig. 2D, and not arrived, *P* < 0.0001, respectively, Fig. 2E). Univariate analysis showed that multiple extranodal involvements, advanced stage, and high levels of serum TCLlnc1 were adverse prognostic factors for both PFS and OS. When using multivariate analysis, high level of serum TCLlnc1 and advanced stage were independent adverse prognostic factors for both PFS and OS (Fig. 2F, G).

**Table 1:** Clinicopathological characteristics of PTCL patients according to TCLlnc1 expression in serum (*n* = 138)^a^.

Variables	Low TCLlnc1 (*n* = 69)	High TCLlnc1 (*n* = 69)	*P* value
Gender			0.4650
Female	20 (29%)	24 (35%)	
Male	49 (71%)	45 (65%)	
Age (years)			0.1677
<60	44 (64%)	36 (52%)	
≥60	25 (36%)	33 (48%)	
ECOG			0.8127
0–1	58 (84%)	59 (86%)	
≥2	11 (16%)	10 (14%)	
LDH level			0.4823
Normal	24 (35%)	28 (41%)	
Elevated	45 (65%)	41 (59%)	
Extranodal involvement			0.0152
<2	48 (70%)	34 (49%)	
≥2	21 (30%)	35 (51%)	
Ann Arbor stage			0.2359
I–II	20 (29%)	14 (20%)	
III–IV	49 (71%)	55 (80%)	
IPI			0.0063
Low risk	45 (65%)	29 (42%)	
High risk	24 (35%)	40 (58%)	
Response to treatment			0.0006
CR	41 (59%)	21 (30%)	
Non-CR	28 (41%)	48 (70%)	

*ECOG* Eastern Cooperative Oncology Group, *LDH* lactic dehydrogenase, *IPI* International Prognostic Index, *CR* complete remission.

^a^*χ*^2^ test.

### TCLlnc1 promotes T-lymphoma cell proliferation and migration in vitro

**Fig. 3:**
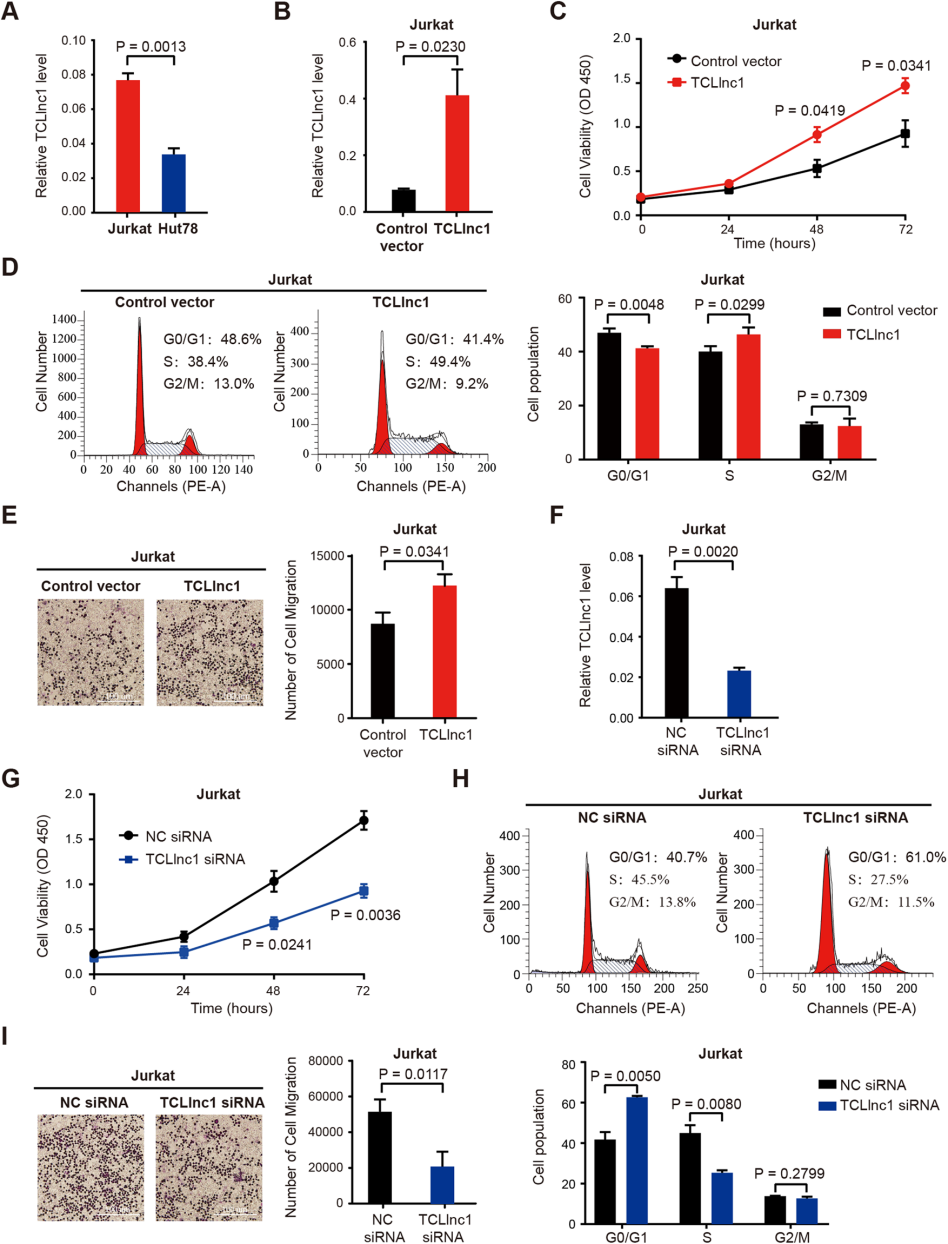
TCLlnc1 promoted T-lymphoma cell proliferation and migration in vitro. **A** Expression of TCLlnc1 in Jurkat and Hut78 cells by qRT-PCR. **B** Overexpression of TCLlnc1 on Jurkat cells. **C**–**E** Effect of TCLlnc1 overexpression on cell proliferation (**C**), cell cycle (**D**), and cell migration (**E**) of Jurkat cells. Representative fields in transwell assay were captured using an inverted microscope (left) and the total number of migrated cells in the lower compartments was counted by flow cytometry (right). The scale bar represents 100 μm. **F** Knockdown of TCLlnc1 on Jurkat cells. **G**–**I** Effect of TCLlnc1 knockdown on cell proliferation (**G**), cell cycle (**H**), and cell migration (**I**) of Jurkat cells. Representative fields in transwell assay were captured using an inverted microscope (left) and the total number of migrated cells in the lower compartments was counted by flow cytometry (right). The scale bar represents 100 μm.

To further determine the biological function of TCLlnc1 in PTCL, overexpression and knockdown of TCLlnc1 were performed using TCLlnc1 plasmid and siRNA on T-lymphoma cell line Jurkat and Hut78. The expression level of TCLlnc1 was measured by qRT-PCR, and Jurkat cells expressed a higher level of TCLlnc1 than those of Hut78 cells (0.076 ± 0.004 vs. 0.033 ± 0.003, *P* = 0.0013, Fig. 3A). Jurkat and Hut78 cells transfected with TCLlnc1 plasmid showed higher expression levels of TCLlnc1 than cells transfected with vectors (Jurkat: 0.401 ± 0.090 vs. 0.076 ± 0.004, *P* = 0.0230, Fig. 3B; Hut78: 0.474 ± 0.038 vs. 0.033 ± 0.003, *P* = 0.0003, Supplementary Fig. 2A). After a 72-h culture, overexpression of TCLlnc1 significantly increased cell proliferation, as compared to vector cells (Jurkat: 1.471 ± 0.085 vs. 0.927 ± 0.150, *P* = 0.0341, Fig. 3C; Hut78: 2.230 ± 0.042 vs. 1.602 ± 0.136, *P* = 0.0116, Supplementary Fig. 2B). Flow cytometric analysis revealed that TCLlnc1 decreased tumor cell arrest in the G0/G1 phase (Jurkat, TCLlnc1 vs. control vector: 41.240 ± 0.413% vs. 46.980 ± 0.927%, *P* = 0.0048; Hut78, TCLlnc1 vs. control vector: 22.810 ± 0.550% vs. 31.170 ± 0.260%, *P* = 0.0002), with an obvious increase in S-phase cells (Jurkat, TCLlnc1 vs. control vector: 46.350 ± 1.524% vs. 40.000 ± 1.176%, *P* = 0.0299, Fig. 3D; Hut78, TCLlnc1 vs. control vector: 51.250 ± 1.407% vs. 46.260% ± 0.806%, *P* = 0.0372, Supplementary Fig. 2C). Meanwhile, overexpression of TCLlnc1 significantly promoted cell migration, as compared to vector cells (Jurkat, 12190 ± 1067 vs. 8672 ± 1060, *P* = 0.0341, Fig. 3E; Hut78, 26,500 ± 4162 vs. 12,702 ± 1179, *P* = 0.0332, Supplementary Fig. 2D). T-lymphoma cells with TCLlnc1 knockdown had significantly lower TCLlnc1 expression levels than siRNA-NC cells (Jurkat, 0.023 ± 0.002 vs. 0.063 ± 0.005, *P* = 0.0020, Fig. 3F; Hut78, 0.005 ± 0.001 vs. 0.014 ± 0.001, *P* = 0.0001, Supplementary Fig. 2E). Knockdown of TCLlnc1 significantly reduced cell proliferation (Jurkat, 0.927 ± 0.076 vs. 1.710 ± 0.104, *P* = 0.0036, Fig. 3G; Hut78, 1.320 ± 0.141 vs. 1.845 ± 0.058, *P* = 0.0261, Supplementary Fig. 2F), decreased S-phase cells (Jurkat, 25.200 ± 1.199% vs. 44.700 ± 3.794%, *P* = 0.0080, Fig. 3H; Hut78, 34.250 ± 1.831% vs. 43.610% ± 1.717%, *P* = 0.0203, Supplementary Fig. 2G) and retarded cell migration (Jurkat, 21,070 ± 8331 vs. 51,844 ± 6899, *P* = 0.0117, Fig. 3I; Hut78, 5647 ± 1150 vs. 13673 ± 2442, *P* = 0.0410, Supplementary Fig. 2H).

### TCLlnc1 promotes T-lymphoma cell proliferation in vivo

**Fig. 4:**
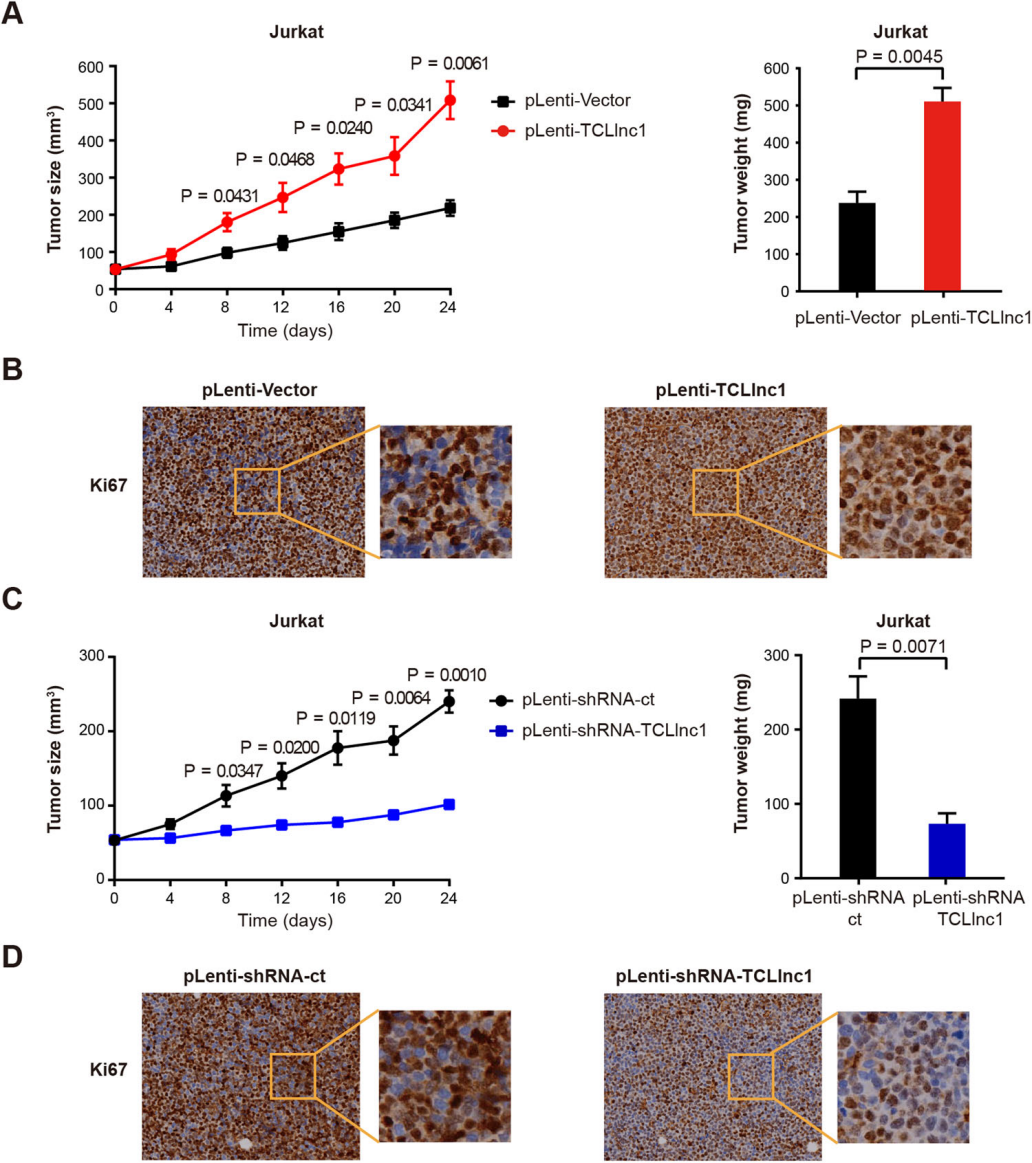
TCLlnc1 promoted T-lymphoma cell proliferation in vivo. **A** Tumor volumes and weights of mice injected with pLenti-Vector- and pLenti-TCLlnc1-transfected Jurkat cells. **B** Immunohistochemistry analysis of Ki-67 in tumors of mice injected with pLenti-Vector- and pLenti-TCLlnc1-transfected Jurkat cells. **C** Tumor volumes and weights of mice injected with pLenti-shRNA-ct- and pLenti-shRNA-TCLlnc1-transfected Jurkat cells. **D** Immunohistochemistry analysis of Ki-67 in tumors of mice injected with pLenti-shRNA-ct- and pLenti-shRNA-TCLlnc1-transfected Jurkat cells.

In order to search for in vivo evidence of TCLlnc1 on lymphoma progression, murine xenograft models were established with subcutaneous injection of Jurkat cells stably transfected with pLenti-TCLlnc1 or pLenti-Vector (as control), as well as pLenti-shRNA-TCLlnc1 or pLenti-shRNA-ct (as control). The sizes and weights of pLenti-TCLlnc1 tumors were significantly larger than those of pLenti-Vector tumors (Fig. 4A). Tumors of pLenti-TCLlnc1 exhibited increased positivity of Ki-67 (Fig. 4B). Accordingly, knockdown of TCLlnc1 reduced tumor growth, thus affecting the weight of pLenti-shRNA-TCLlnc1 tumors, as compared to pLenti-shRNA-ct tumors (Fig. 4C). Tumors of pLenti-shRNA-TCLlnc1 exhibited decreased positivity of Ki-67 (Fig. 4D).

### TCLlnc1 activates tumor growth factor (TGF-β) signaling and regulates cytokine production

**Fig. 5:**
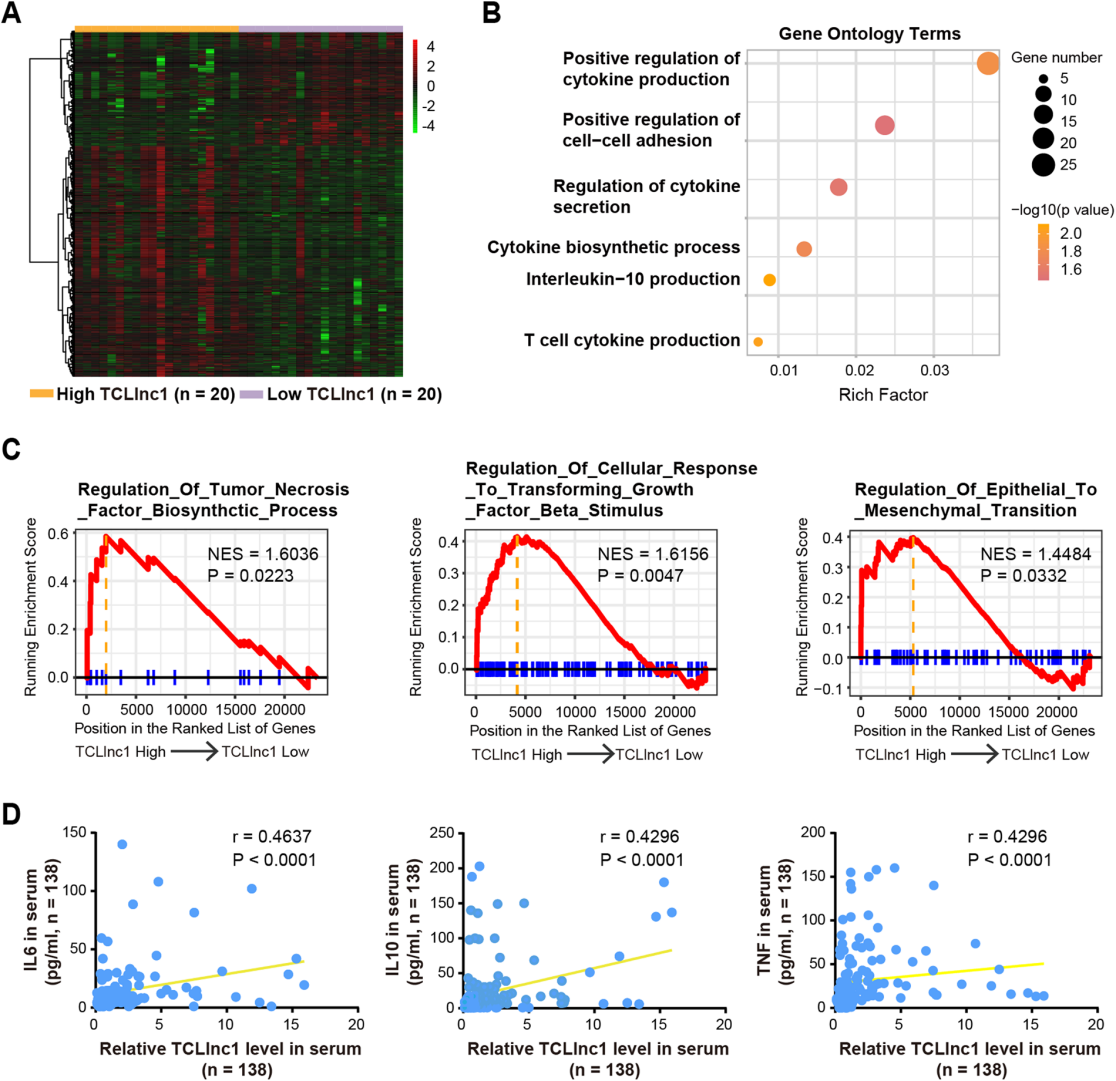
TCLlnc1 activated TGF-β signaling and increased cytokine production. **A** RNA-seq data of tumors of PTCL patients (*n* = 40) according to TCLlnc1 expression. **B** Gene ontology terms of differentially expressed genes according to TCLlnc1 expression in tumors. **C** Gene set enrichment analysis (GSEA) according to TCLlnc1 expression in tumors. **D** Correlation of TCLlnc1 expression with cytokines levels in serum of PTCL patients (*n* = 138).

To better understand the underlying molecular mechanism of TCLlnc1, we performed RNA-seq analysis to obtain the transcriptional profiles in tumor samples of PTCL patients according to TCLlnc1 expression. Forty PTCL patients were divided into high- and low-expression groups based on the median value of TCLlnc1. A total of 1115 differentially expressed transcripts (fold change ≥1.5, *P* < 0.05) were identified; these included 372 upregulated and 743 downregulated transcripts (Fig. 5A). Gene ontology (GO) analysis showed that the most significantly altered biological processes included pathways involved in cytokine production, cell–cell adhesion, and cytokine secretion (Fig. 5B). Gene set enrichment analysis (GSEA) revealed that the gene sets were significantly related to regulation of tumor necrosis factor (TNF) biosynthetic process, cellular response to TGF-β stimulus, and regulation of epithelial to mesenchymal transition (Fig. 5C). Clinically, the correlation between the expression levels of TCLlnc1 and cytokines, such as interleukin-6 (IL-6), IL-10, and TNF in serum (Fig. 5D) of PTCL patients indicated that TCLlnc1 activated TGF-β signaling pathway and regulated cytokine production in PTCL.

### TCLlnc1 interacts with HNRNPD and YBX1 as a modular scaffold

**Fig. 6:**
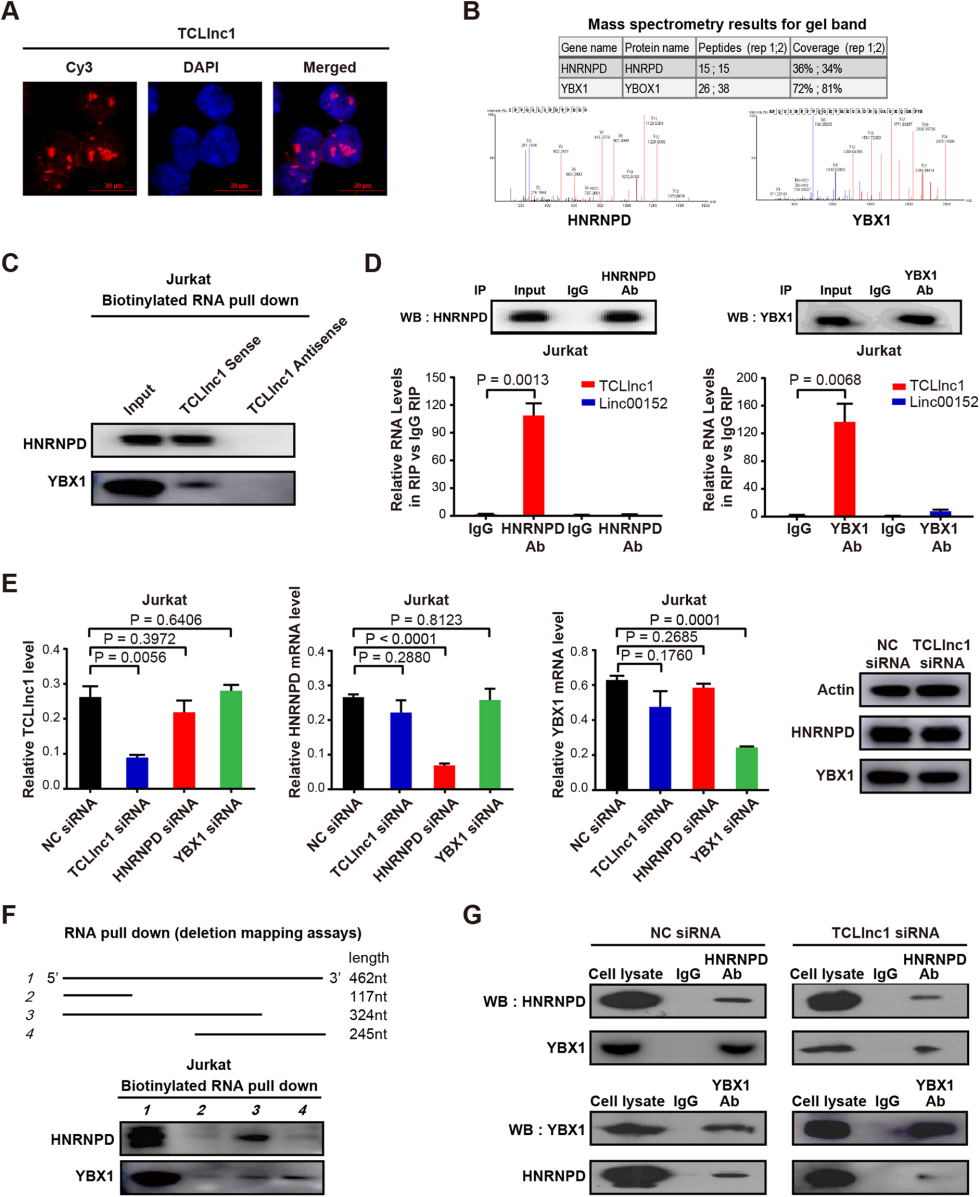
TCLlnc1 physically interacted with heterogeneous nuclear ribonucleoprotein D (HNRNPD) and Y-box binding protein-1 (YBX1). **A** Nuclear location of TCLlnc1 in Jurkat cells (red). Nuclei were stained by 4′, 6-diamidino-2-phenylindole (DAPI, blue). **B** TCLlnc1-binding proteins detected by mass spectrometry. **C** Western blot validation of proteins pulled down with TCLlnc1. **D** Interaction of TCLlnc1 with HNRNPD and YBX1 detected by RNA immunoprecipitation (RIP) assay. The fold enrichment of TCLlnc1 in RIP is relative to its matching IgG control RIP. LncRNA linc00152 was referred as a negative control. **E** Expression of TCLlnc1, HNRNPD, and YBX1 after indicated RNA knockdown detected by qRT-PCR and western blot. **F** Western blot analysis of HNRNPD and YBX1 pulled down by full-length or truncated TCLlnc1. **G** Interaction of HNRNPD and YBX1 after TCLlnc1 knockdown detected by co-immunoprecipitation (co-IP).

LncRNA exerts biological function by physically interacting with transcriptional factors, histone regulators, and other cellular factors^35^. FISH assay showed that TCLlnc1 was mainly located in the nucleus of Jurkat cells (Fig. 6A), suggesting that TCLlnc1 may function at the transcriptional level via interacting with nucleus molecules or proteins. To further explore the underlying molecular mechanism, biotinylated TCLlnc1 and antisense TCLlnc1 RNA (negative control) were incubated with total protein extracts from Jurkat cells and pulled down with streptavidin. Only HNRNPD and YBX1 were detected by mass spectrometry from three independent RNA pull-down assays (Fig. 6B) and immunoblotting (Fig. 6C).

Physical interaction of TCLlnc1 with HNRNPD and YBX1 was validated by RIP using anti-HNRNPD antibody and anti-YBX1 antibody, with linc00152 as a negative control. TCLlnc1 is specifically bound to HNRNPD and YBX1 in Jurkat cells (Fig. 6D). In addition, qRT-PCR and western blot assays showed that knockdown of TCLlnc1 had no effect on HNRNPD and YBX1 mRNA expression or protein expression. The knockdown of HNRNPD and YBX1 had no effect on TCLlnc1 expression (Fig. 6E). These data indicated that TCLlnc1 physically interacted with HNRNPD and YBX1. Subsequently, a series of TCLlnc1 deletion mutants were constructed to map the precise binding regions for HNRNPD and YBX1. According to in vitro RNA pull-down assays, segment 1–324 nt of TCLlnc1 interacted with HNRNPD, while segment 216–462 nt of TCLlnc1 interacted with YBX1 (Fig. 6F). Moreover, as revealed by co-IP, HNRNPD, and YBX1 interacted with each other in Jurkat cells and could be attenuated by knockdown of TCLlnc1 (Fig. 6G). Together, TCLlnc1 was important for HNRNPD and YBX1 complex formation as a modular scaffold.

### TCLlnc1 functions with HNRNPD and YBX1 complex in regulating TGF-β signaling pathway

**Fig. 7:**
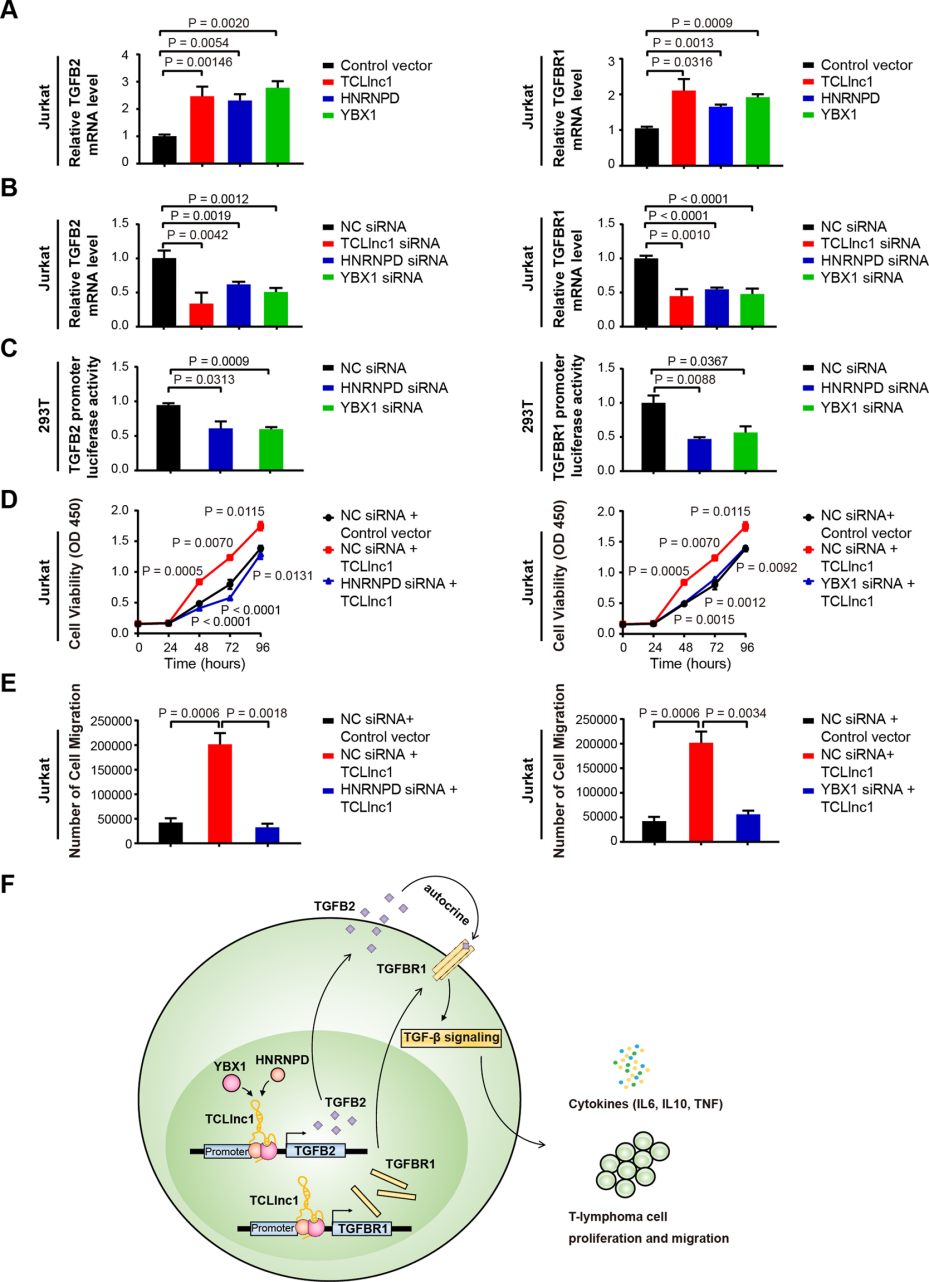
TCLlnc1 provoked lymphoma progression via HNRNPD and YBX1. **A** Expression of *TGFB2* and *TGFBR1* after overexpression of TCLlnc1, HNRNPD, or YBX1 detected by qRT-PCR. **B** Expression of *TGFB2* and *TGFBR1* after knockdown of TCLlnc1, HNRNPD, or YBX1 detected by qRT-PCR. **C** Luciferase activity assay of *TGFB2* and *TGFBR1* promoter in cells transfected with HNRNPD siRNA, YBX1 siRNA, or NC siRNA. **D** Proliferation of Jurkat cells after transfection of HNRNPD siRNA or the YBX1 siRNA, plasmid encoding TCLlnc1, or control vector. **E** Migration of Jurkat cells after transfection of HNRNPD siRNA or YBX1 siRNA, plasmid encoding TCLlnc1, or control vector. **F** A schematic model of lncRNA TCLlnc1 function in PTCL.

Because lncRNA regulates downstream genes expression by interacting with transcription factors, we next investigated which of the TGF-β signaling pathway genes are regulated by the TCLlnc1, HNRNPD and YBX1 complex. As shown by qRT-PCR in Jurkat cells, the relative mRNA expression level of *TGF-β2* (*TGFB2*) and *TGF-β receptor I* (*TGFBR1*) was significantly upregulated by overexpression of TCLlnc1 (*TGFB2*: 1.004 ± 0.064 vs. 2.471 ± 0.350, *P* = 0.0146; *TGFBR1*: 1.049 ± 0.044 vs. 2.105 ± 0.323, *P* = 0.0316), HNRNPD (*TGFB2*: 1.004 ± 0.064 vs. 2.311 ± 0.229, *P* = 0.0054; *TGFBR1*: 1.049 ± 0.044 vs. 1.653 ± 0.061, *P* = 0.0013), and YBX1 (*TGFB2*: 1.004 ± 0.064 vs. 2.777 ± 0.240, *P* = 0.0020; *TGFBR1*: 1.049 ± 0.044 vs. 1.920 ± 0.087, *P* = 0.0009) (Fig. 7A), but was downregulated by knockdown of TCLlnc1 (*TGFB2*: 1.004 ± 0.065 vs. 0.338 ± 0.093, *P* = 0.0042; *TGFBR1*: 1.000 ± 0.225 vs. 0.446 ± 0.060, *P* = 0.0010), HNRNPD (*TGFB2*: 1.004 ± 0.065 vs. 0.620 ± 0.023, *P* = 0.0051; *TGFBR1*: 1.000 ± 0.225 vs. 0.548 ± 0.016, *P* < 0.0001), and YBX1 (*TGFB2*: 1.004 ± 0.065 vs. 0.509 ± 0.034, *P* = 0.0025; *TGFBR1*: 1.000 ± 0.225 vs. 0.4777 ± 0.047, *P* = 0.0006) (Fig. 7B). Other key genes of TGF-β signaling pathway were not altered by TCLlnc1, HNRNPD, or YBX1, such as *TGF-β1* (*TGFB1*), *TGF-β3* (*TGFB3*), *TGF-β receptor II* (*TGFBR2*), and *SMAD2*, *SMAD3*, *SMAD4* (Supplementary Fig. 3A). qRT-PCR analysis of *TGFB2* and *TGFBR1* expression was performed in the pLenti-Vector, pLenti-TCLlnc1-, pLenti-shRNA-NC-, and pLenti-shRNA-TCLlnc1-transfected Jurkat cells, revealing similar results in vivo (Supplementary Fig. 3B).

To finally confirm the direct regulation of TCLlnc1/HNRNPD/YBX1 complex to *TGFB2* and *TGFBR1* expression, luciferase assays were performed. As expected, HNRNPD and YBX1 activated *TGFB2* and *TGFBR1* transcription by binding to the promoters of *TGFB2* and *TGFBR1* (Fig. 7C). The knockdown of HNRNPD or YBX1 reversed TCLlnc1-mediated cell proliferation (Fig. 7D) and migration (Fig. 7E). These data demonstrated that TCLlnc1 exerted its function by binding to HNRNPD and YBX1. TCLlnc1/HNRNPD/YBX1 complex regulated key gene transcription to activate TGF-β signaling pathway, induce cytokine production, and promote cell proliferation and invasion in PTCL (Fig. 7F).

## Discussion

LncRNAs are aberrantly expressed in various cancers and actively participate in disease progression^12^. In the present study, TCLlnc1 was identified as a lncRNA overexpressed in the main PTCL histological subtypes linked to both lymphoma cell proliferation and in vitro and in vivo migration. Of note, circulating lncRNAs have potential utility as noninvasive and blood-based biomarkers^36^. Accordingly, we revealed that serum TCLlnc1 expression correlated with tumor TCLlnc1 expression and was significantly associated with extranodal involvement, high-risk IPI, and independently indicated poor patients’ clinical outcomes. Therefore, not only do our findings support the essential role TCLlnc1 plays during tumor progression, but we also provide a potential biomarker for PTCL disease prognosis prediction.

TGF-β signaling pathway represents one of the best-recognized cascades modulating tumor proliferation and migration^37^. We showed that TCLlnc1 overexpression led to the activation of TGF-β signaling pathway and production of cytokines, such as IL-6, IL-10, and TNF. The results are consistent with the previous study regarding cutaneous T cell lymphoma, stating that TGF-β and IL-10 are hallmarks of advanced stages, increased cell migration, and decreased antitumor responses^38^. TGF-β is composed of three isoforms, TGFB1, TGFB2, and TGFB3. In order to activate the TGF-β signaling pathway, TGF-β isoforms bind to TGFBR2, thereby recruiting TGFBR1 and upregulate downstream cascade^37^. Here, *TGFB2* and *TGFBR1* were potential targets of TGF-β signaling activation provoked by TCLlnc1 overexpression. Galuniertib, a small-molecular inhibitor of TGFBR1, has recently been reported to possess efficient tumor inhibitory activity in breast cancer^39^ and multiple myeloma^40^. Based on the fact that TCLlnc1 promoted both tumor growth and the migration of PTCL via TGF-β signaling activation, therapeutic targeting of TGF-β may counteract tumor progression in PTCL with TCLlnc1 overexpression.

LncRNAs interact with RNA-binding proteins to form nuclear ribonucleoprotein complexes, which are crucial regulators of transcriptional programs in the nucleus^41,42^. Our results demonstrated that TCLlnc1 directly interacted with HNRNPD and YBX1 and acts as a modular scaffold in PTCL. HNRNPs represent an RNA-binding ribonucleoprotein family of transcription activators that are implicated in RNA stability and gene expression regulation in various cancers^41^. In colorectal cancer, HNRNPD interacts with LINC01354 and contributes to proliferation and metastasis through the activation of the Wnt/β-catenin signaling pathway^43^. YBX1 is also a transcription activator of the RNA- and DNA-binding domain. The RNA-binding domain can bind to lncRNA or mRNA, whereas the DNA-binding domain binds to a consensus sequence in the target genes promoter, implicating numerous cellular processes, particularly tumorigenesis^44^. More importantly, in multiple cancer types, S-phase-enriched lncRNAs interact with HNRNPK–YBX1 complex and affect cancer cell hallmarks through regulating phosphatidylinositol-3-kinase/AKT and MAPK signaling activation^45^. Our findings indicated that HNRNPD and YBX1 are required for TCLlnc1-mediated regulation of TGF-β signaling pathway, as well as T-lymphoma cell proliferation and migration. The results further strengthen the understanding of lncRNA as an oncogenic driver on PTCL progression.

## Conclusions

TCLlnc1 is a clinically, functionally, and mechanistically oncogenic lncRNA in PTCL. TCLlnc1 and its downstream signaling pathway may be meaningful for risk stratification and targeted therapy in patients with PTCL.

## Supplementary information

Supplementary Tables

Supplementary Figure legends

Supplementary Figure 1

Supplementary Figure 2

Supplementary Figure 3

## Data Availability

The datasets used and/or analyzed during the current study are available from the corresponding author on reasonable request.
