# Supplementary material for: A novel lncRNA TCLlnc1 promotes peripheral T cell lymphoma progression through acting as a modular scaffold of HNRNPD and YBX1 complexes
Source: Cell Death Dis. 2021 Mar 25;12(4):321. doi: 10.1038/s41419-021-03594-y (PMC7994313; doi:10.1038/s41419-021-03594-y)
Supplement: Supplementary file 1 — Supplementary Tables [file 41419_2021_3594_MOESM1_ESM.docx]

| Characteristics | N = 138 |
| --- | --- |
| Gender |  |
| Female | 44 (32%) |
| Male | 94 (68%) |
| Age (y) |  |
| < 60 | 80 (58%) |
| ≥60 | 58 (42%) |
| ECOG |  |
| 0-1 | 117 (85%) |
| ≥2 | 21 (15%) |
| LDH level |  |
| Normal | 52 (38%) |
| Elevated | 86 (62%) |
| Extranodal involvement |  |
| < 2 | 82 (59%) |
| ≥ 2 | 56 (41%) |
| Ann Arbor stage |  |
| I to II | 34 (25%) |
| III to Ⅳ | 104 (75%) |
| IPI |  |
| Low risk | 74 (54%) |
| High risk | 64 (46%) |
| Response to treatment |  |
| CR | 62 (45%) |
| Non-CR | 76 (55%) |
| Race  Chinese  Others | 138 (100%)  0 |

Supplementary Table 1. Clinicopathological characteristics of PTCL patients (n = 138)

Abbreviations: ECOG, Eastern Cooperative Oncology Group; LDH, lactic dehydrogenase; IPI, International Prognostic Index; CR, complete remission.

Supplementary Table 2. Primer sequences for qRT-PCR

| Target gene | Forward Primer (5' to 3') | Reverse Primer (5' to 3') |
| --- | --- | --- |
| TCLlnc1 | AGGTGGCAGAATCTTGCTTCGG | GGCCAGTTTGCACAGTTCCAGT |
| HNRNPD | GCGTGGGTTCTGCTTTATTACC | TTGCTGATATTGTTCCTTCGACA |
| YBX1 | GGACAAGAAGGTCATCGCAAC | TCTCCATCTCCTACACTGCGA |
| TGFB2 | CAGCACACTCGATATGGACCA | CCTCGGGCTCAGGATAGTCT |
| TGFBR1 | CACAGAGTGGGAACAAAAAGGT | CCAATGGAACATCGTCGAGCA |

Supplementary Table 3. SiRNA sequences

| siRNA | Sequences (5' to 3') |
| --- | --- |
| TCLlnc1 siRNA | GGAGACAGTGATGACAAGA |
| HNRNPD siRNA | AGACUGCACUCUUGAAGUUATT |
| YBX1 siRNA | CCACGCAATTACCAGCAAA |
| Control siRNA | UUCUCCGAACGUGUCACGUTT |
