# Supplementary material for: A novel lncRNA TCLlnc1 promotes peripheral T cell lymphoma progression through acting as a modular scaffold of HNRNPD and YBX1 complexes
Source: Cell Death Dis. 2021 Mar 25;12(4):321. doi: 10.1038/s41419-021-03594-y (PMC7994313; doi:10.1038/s41419-021-03594-y)
Supplement: Supplementary file 2 — Supplementary Figure legends [file 41419_2021_3594_MOESM2_ESM.docx]

**Supplementary Figure 1. TCLlnc1 was non-coding RNA.** **A** Analysis of lncRNAs-oncogenes coexpression in reactive hyperplasia (RH) and peripheral T-cell lymphoma (PTCL). **B** Expression of five candidate lncRNAs detected by quantitative real-time polymerase chain reaction (qRT-PCR) in PTCL (n = 10) and RH (n = 10). **C** Coding Potential Assessment Tool (CPAT) and Coding Potential Calculator (CPC) were used to assess the coding potential of TCLlnc1 and other known coding and non-coding RNAs.

**Supplementary Figure 2.** **TCLlnc1 enhanced T-lymphoma cell proliferation and migration in vitro.** **A** Overexpression of TCLlnc1 on Hut78 cells. **B-D** Effect of TCLlnc1 overexpression on cell proliferation (B), cell cycle (C) and cell migration (D) of Hut78 cells. The scale bar represents 50 μm. **E** Knockdown of TCLlnc1 on Hut78 cells. **F-H** Effect of TCLlnc1 knockdown on cell proliferation (F), cell cycle (G) and cell migration (H) of Hut78 cells. The scale bar represents 50 μm.

**Supplementary Figure 3.** **Impact of TCLlnc1, Heterogeneous nuclear ribonucleoprotein D (HNRNPD) or Y-box binding protein-1 (YBX1) expression on key genes of TGF-β signaling pathway.** **A** Expression levels of genes detected by qRT-PCR in Jurkat cells after knockdown of TCLlnc1, HNRNPD or YBX1. **B** Expression level of *TGFB2* and *TGFBR1* detected by qRT-PCR in murine tumors transfected with pLenti-Vector, pLenti-TCLlnc1, pLenti-shRNA-ct, and pLenti-shRNA-TCLlnc1.
