# Supplementary figures and images for: A novel lncRNA TCLlnc1 promotes peripheral T cell lymphoma progression through acting as a modular scaffold of HNRNPD and YBX1 complexes
Source: Cell Death Dis. 2021 Mar 25;12(4):321. doi: 10.1038/s41419-021-03594-y (PMC7994313; doi:10.1038/s41419-021-03594-y)

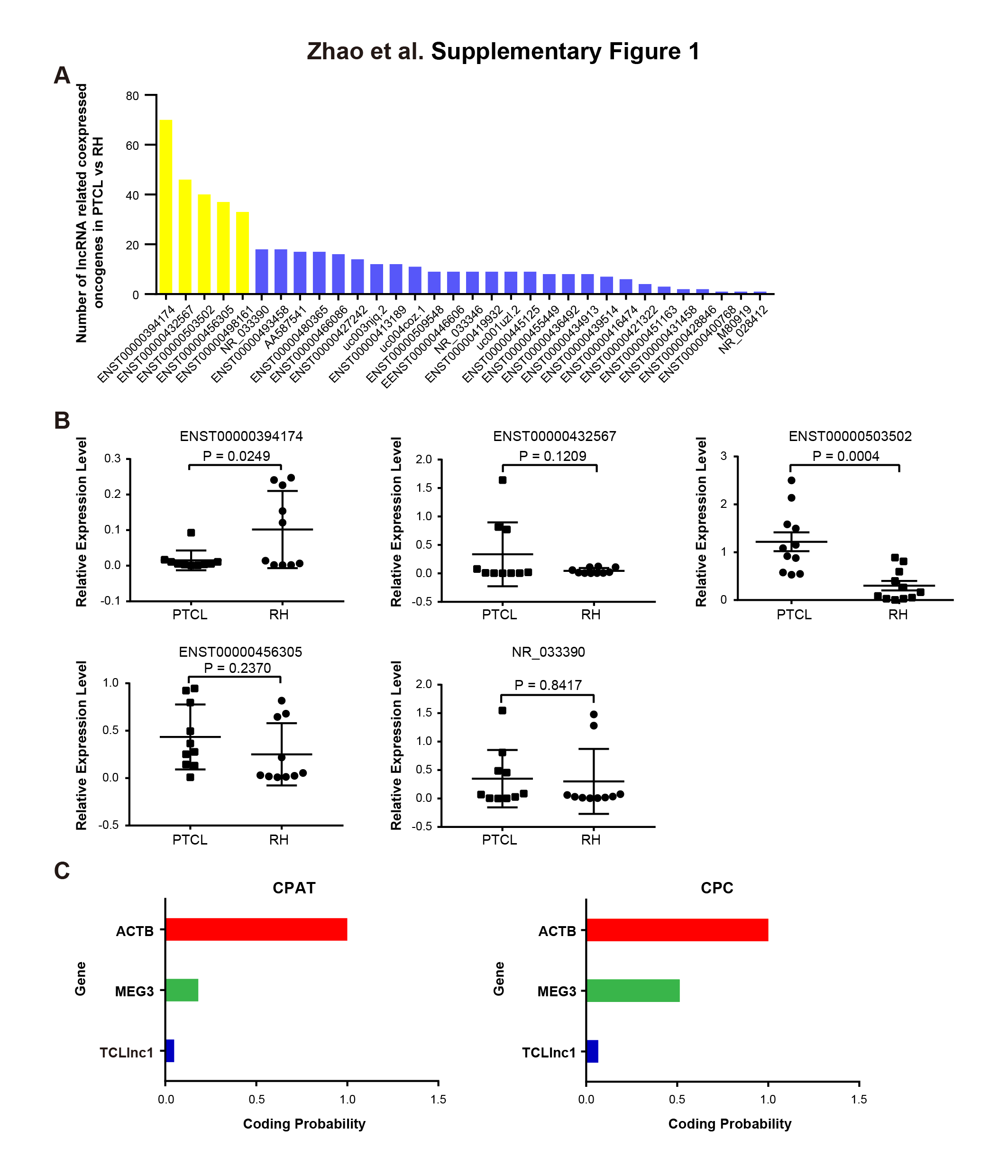

Supplement: Supplementary file 3 — Supplementary Figure 1 [file 41419_2021_3594_MOESM3_ESM.png]

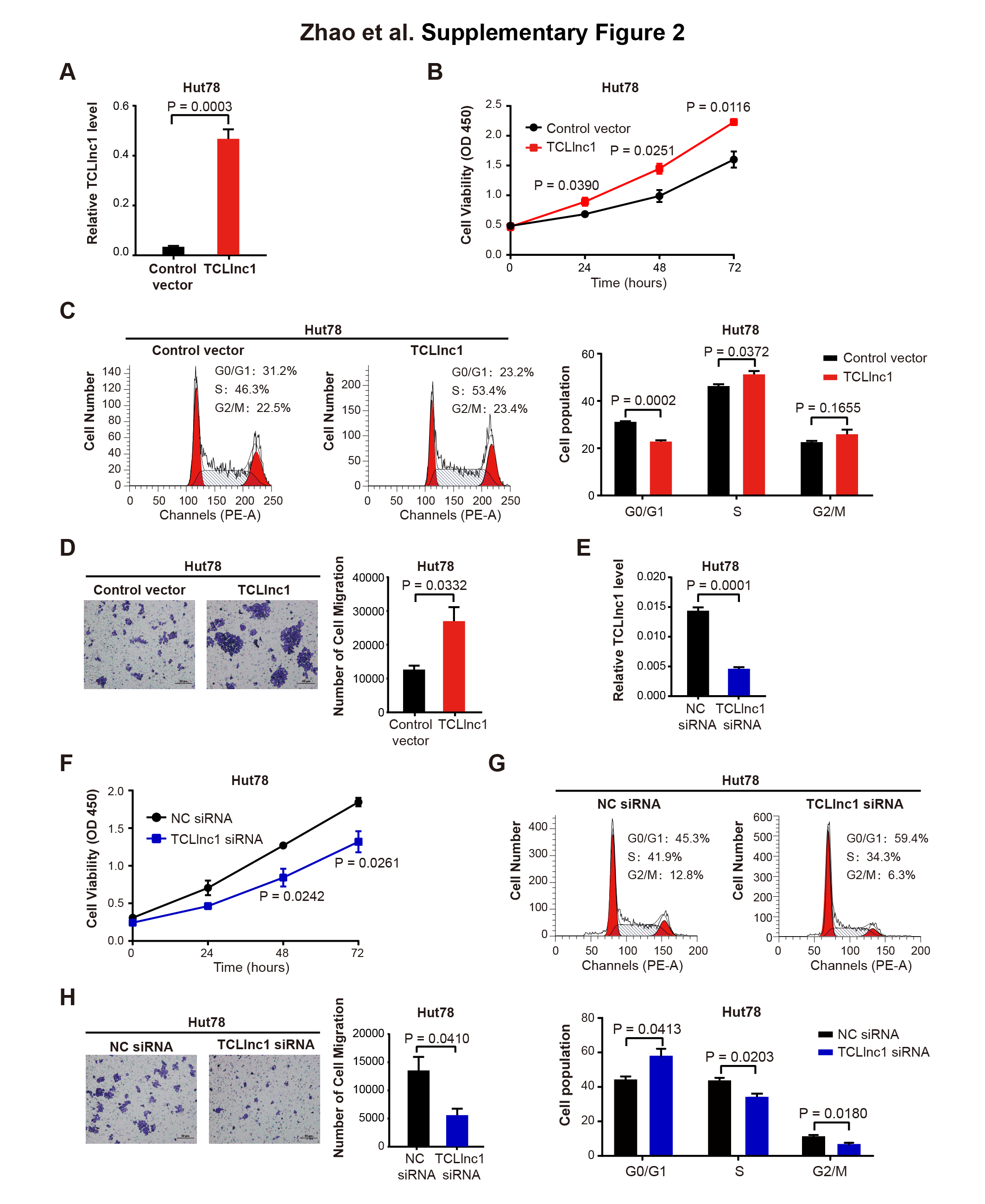

Supplement: Supplementary file 4 — Supplementary Figure 2 [file 41419_2021_3594_MOESM4_ESM.png]

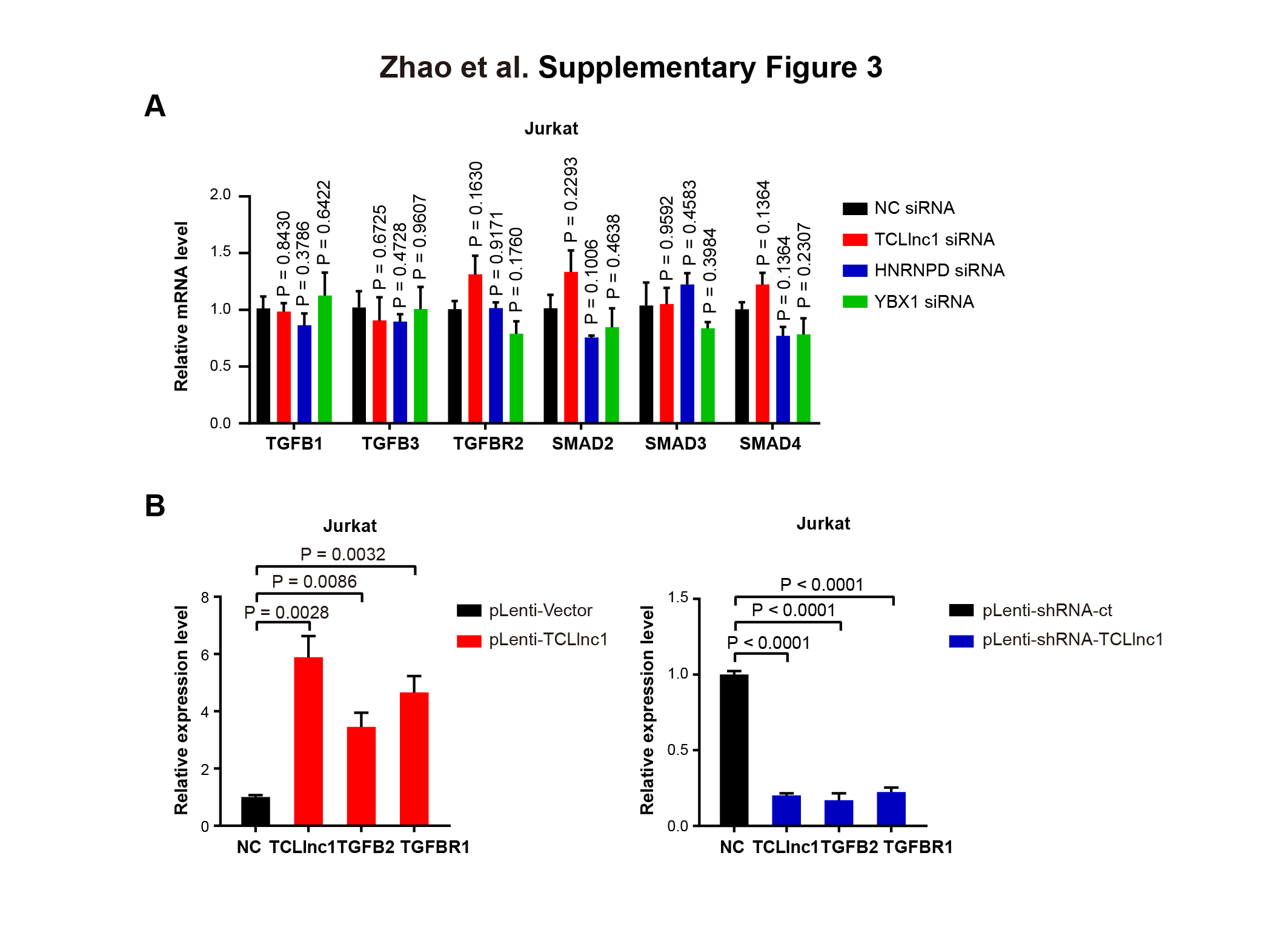

Supplement: Supplementary file 5 — Supplementary Figure 3 [file 41419_2021_3594_MOESM5_ESM.png]
